# Novel Syndrome With Congenital Thumb Aplasia, Epilepsy, Cognitive Impairment, and Myopathy: A Case Report

**DOI:** 10.7759/cureus.76302

**Published:** 2024-12-24

**Authors:** Josef Finsterer, Awini Barwari

**Affiliations:** 1 Neurology, Neurology and Neurophysiology Center, Vienna, AUT

**Keywords:** chromosomal defect, cognitive decline, developmental delay, epilepsy, thumb aplasia

## Abstract

The combination of thumb aplasia, epilepsy, cognitive impairment, skeletal deformities, and myopathy has not been previously reported. The patient is a 22-year-old man with congenital bilateral thumb aplasia, developmental delay, and cognitive impairment who suffered a first tonic-clonic seizure at the age of 16 and was treated with valproic acid (VPA). At the age of 22, lamotrigine was added due to seizure recurrences and absences. Neurological examination revealed cognitive impairment, latent strabismus, thumb aplasia, orthopedic anomalies of the feet, gnome calves, and absent tendon reflexes. Electromyography indicated a myopathy. Although the family history was negative for thumb aplasia, epilepsy, mental retardation, myopathy, and orthopedic disease, the clinical presentation of the index patient was suspected due to a sporadic genetic defect at either the chromosomal or gene level. Chromosomal micro- or macrodeletions or duplications as well as point mutations in PTPRQ, SALL4, RECQL4, and SALL1 have previously been identified in syndromic thumb aplasia. In conclusion, the presented case represents a novel syndrome with the phenotypic manifestations of epilepsy, myopathy, cognitive impairment, mild dysmorphism, and congenital thumb aplasia. Physicians should remain vigilant and interested in patients with syndromic thumb aplasia.

## Introduction

Congenital thumb aplasia has only rarely been reported. It occurs either as an isolated phenomenon (non-syndromic thumb aplasia) or in combination with other phenotypic features (syndromic thumb aplasia) [1-3]. Thumb aplasia together with epilepsy has not been reported; however, thumb hypoplasia or thumb deformities together with epilepsy have been reported in carriers of microdeletions, encompassing the loci of the SCN3A, SCN2A, TTC21B, SCN1A, and SCN9A genes [4], or of point mutations in KCNH1 that manifest phenotypically as Zimmermann-Laband syndrome or Temple-Baraitser syndrome [5]. Other disorders that go along with thumb deformities include the Larsen of La Reunion Island syndrome, ring-chromosome 4 syndrome, thumb agenesis due to microduplication of 22q11.21, thumb agenesis and radioulnar synostosis due to mutations in SALL4, Rothmund-Thomson syndrome, and Baller-Gerold syndrome. These disorders can also be associated with intellectual disability [5]. Here, we present a patient with a combination of congenital thumb aplasia, epilepsy, cognitive impairment, skeletal deformities, and myopathy. He also had developmental delay and mild dysmorphism. The combination of these features has not been reported before.

## Case presentation

The patient is a 22-year-old man who was diagnosed with bilateral thumb aplasia (thumb agenesis) at birth. He was later diagnosed with delayed walking until the age of three, bedwetting until the age of six, and cognitive impairments. Despite these deficits, he managed to complete primary and secondary school with the support of caregivers and the relevant authorities. He himself did not complain of pain, cramps, easy tiring, muscle weakness, auras, or sensory disturbances. At the age of 16, he suffered a first tonic-clonic seizure (TCS), which is why valproic acid (VPA) (1000 mg/d) was started. After suffering his second TCS a few months later, the VPA was increased to 1500 mg/d. After the third TCS a year later, the VPA was increased to 2000 mg/d. He has had no further TCSs since then, but his mother noticed recurrent absences with increasing frequency in the last few months before the presentation. She also reported that her son had experienced a prolonged recovery period after general anesthesia at 1.5 years of age. The family history was negative for epilepsy, and there was no previous traumatic brain injury (TBI), encephalitis, meningitis, birth trauma, or febrile convulsions in childhood in the index patient. None of the other first-degree family members had epilepsy, thumb aplasia, myopathy, or cognitive impairment.

**Figure 1:**
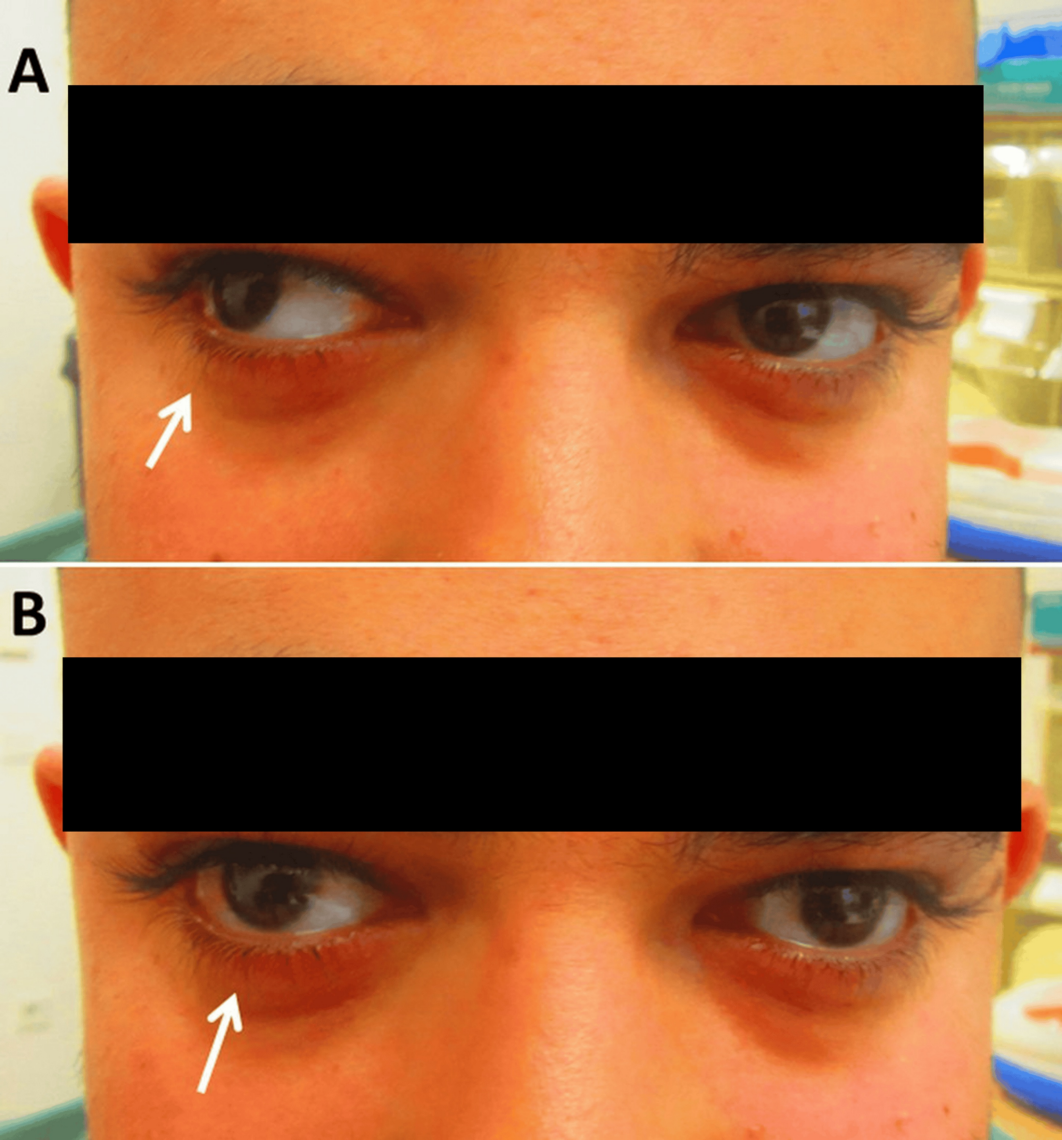
Delayed adduction of the right bulb when looking from right to left in the horizontal plane. At the beginning of adduction, the right bulb remained in the abduction position (panel A). Both bulbs were almost parallel when they reached the midline (panel B)

**Figure 2:**
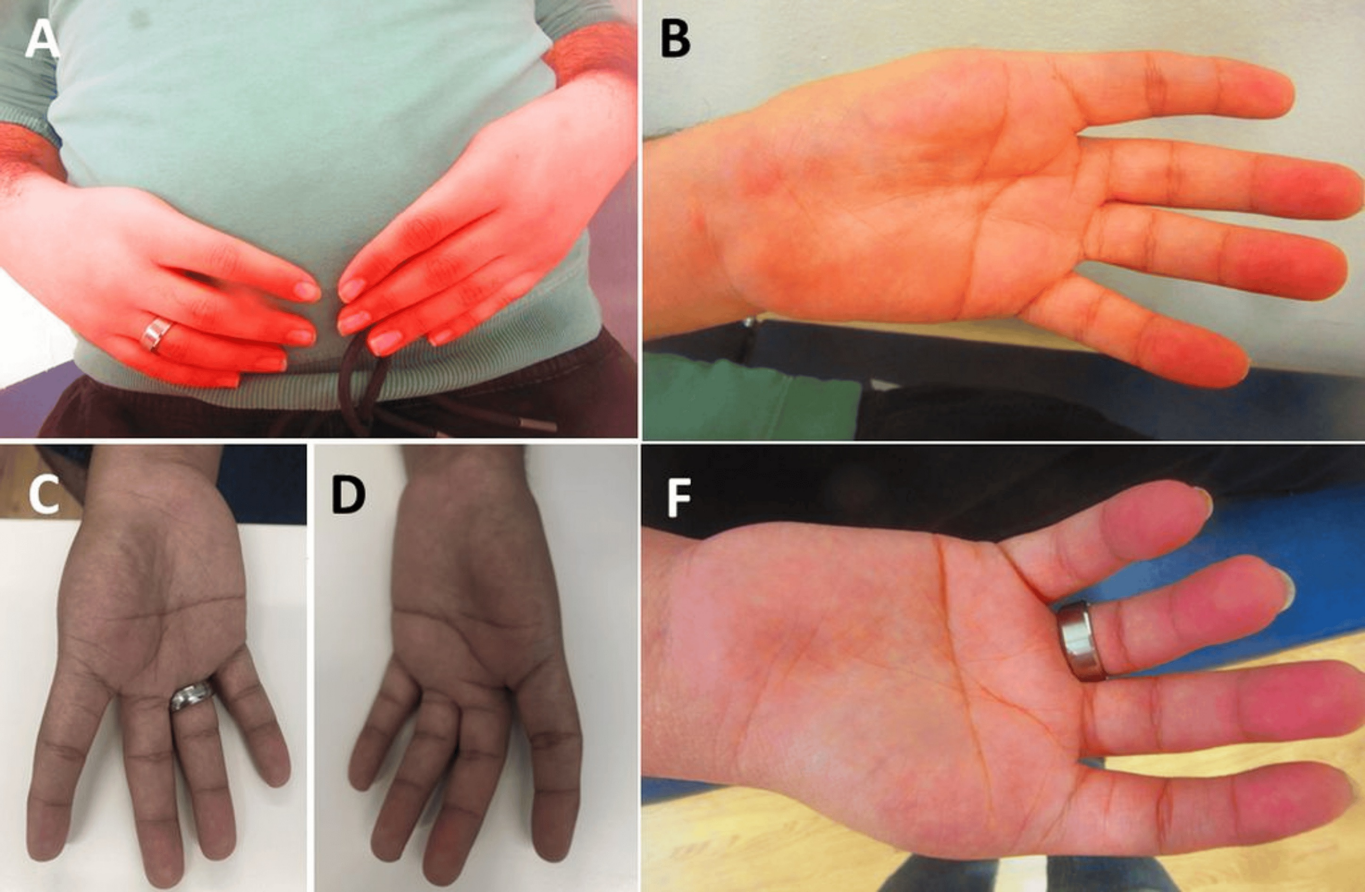
Aplasia of both thumbs with normal morphology of the remaining four fingers. Both hands in pronation (panel A). Left hand in supination (panels B and D). Right hand in supination (panels C and E)

**Figure 3:**
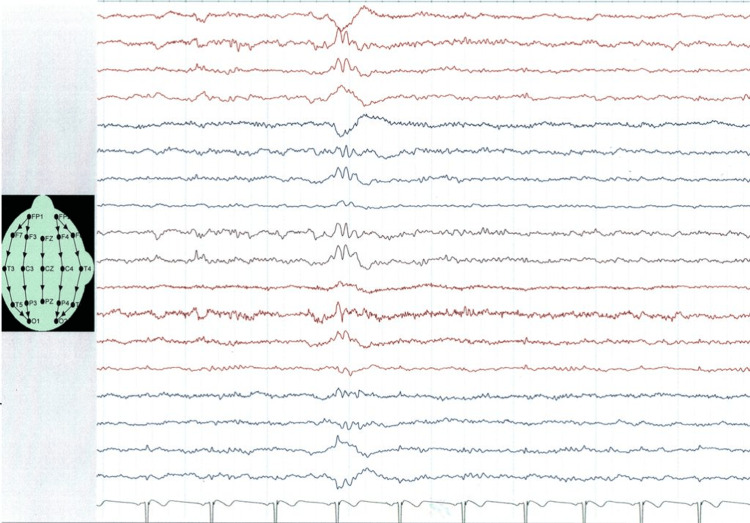
EEG recordings at the age of 22 showing short episodes of focal and generalized sharp waves EEG: electroencephalography

**Figure 4:**
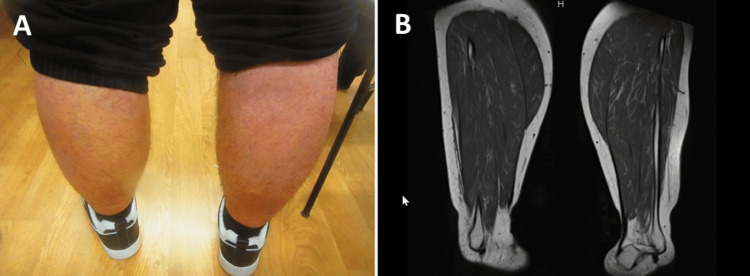
Dwarf calves without fatty or fibrous degeneration on assessment (panel A) and on muscle MRI (panel B) MRI: magnetic resonance imaging

Clinical neurologic examination revealed a shy, taciturn nature with intellectual disabilities without facial dysmorphism but delayed adduction of the right bulb when looking from the lateral position to the orthotopic position (Figure 1), aplasia of both thumbs (Figure 2), hypermobile joints, with no history of dislocations or subdislocations, gnome calves, outward pointing feet, and inversion position of the feet and generally decreased tendon reflexes. He communicated and played with his friends without problems, was able to perform activities of daily living without major limitations, and denied pain or any motor or sensory disturbances. Creatine kinase was at the upper normal range, but lactate was normal. Triglycerides, cholesterol, and liver enzymes were mildly elevated, but all other routine blood parameters were within the normal range. Nerve conduction studies ruled out peripheral large fiber neuropathy, but needle electromyography indicated myopathy. Electroencephalography (EEG) showed epileptiform discharges such as episodes of generalized, slow, polymorphic sharp waves lasting up to two seconds (Figure 3). Magnetic resonance imaging (MRI) of the brain was inconclusive. MRI of the muscles was normal, with the exception of the bilateral calves (Figure 4). It was recommended that he be tested for chromosomal abnormalities, but the parents have not yet agreed to this test. Since VPA caused generalized fatigue and exhaustion, abnormal EEG, and the questionable absence seizures, at the age of 22 years, lamotrigine 100mg/d was added. With this therapy, absences and tonic-clonic seizures disappeared completely.

## Discussion

The patient presented is of interest for a novel thumb aplasia syndrome, most likely due to a sporadic mutation or chromosomal defect, as no other first-degree family members had a similar phenotype. The patient was hardly disabled by the missing thumbs and experienced little restriction in quality of life or range of daily activities. He had learned to compensate for his motor deficits and was still able to play sports and work as a truck mechanic. At the age of 22, he was still living with his mother and other siblings and was able to participate sufficiently in housework. The epilepsy, which had developed since the age of 16, was adequately controlled with a combination therapy of VPA (2000 mg/d) and lamotrigine (100 mg/d). The myopathy manifested in the form of gnome calves and adduction insufficiency of the right bulb. The patient also had cognitive impairment.

The presented combination of phenotypic characteristics has not yet been reported. However, there are a few reports of thumb deformities and epilepsy [4] and thumb deformities and mental retardation [6]. In Townes-Brocks syndrome, thumb deformity has been reported together with abnormal foot postures due to heterozygous pathogenic variants in SALL1 [7]. The protein encoded by SALL1 is a zinc finger transcriptional repressor and belongs to the NuRD histone deacetylase complex (HDAC). Two transcript variants encoding different isoforms have been found for this gene. In addition to Townes-Brocks syndrome, mutations in SALL1 cause bronchio-oto-renal syndrome (BOR). Thumb aplasia along with arthrogryposis was reported in a 42-year-old woman with Edwards-Klinefelter syndrome due to a double aneuploidy (48, XXY +18) [8]. Thumb aplasia has also been reported in association with cleft lip and congenital cardiac anomalies (e.g., aortopulmonary window) [9]. The absence of the thumb and radius has also been reported in association with tracheoesophageal fistula and ventricular septal defect as part of the VACTERL complex [10]. Aplasia of the thumbs can also be a phenotypic feature of trisomy 18, which also manifests with holoprosencephaly, ventricular septal defect, and arthrogryposis of the bilateral wrists [11]. Thumb malformations along with other organ abnormalities may also be a feature of Townes-Brocks syndrome, which is due to pathogenic variants in PTPRQ and SALL1 [12]. PTPRQ encodes a member of the receptor-like protein tyrosine phosphatase type III family. The encoded protein catalyzes the dephosphorylation of phosphotyrosine and phosphatidylinositol and plays a role in cell proliferation and differentiation. Mutations at this locus have also been associated with autosomal recessive deafness. Thumb agenesis was also found in a patient with a microduplication of 22q11.21 [13]. In a patient with Holt-Oram syndrome and thumb agenesis, the phenotype was attributed to a variant in SALL4 [14]. SALL4 is a transcription factor encoded by a member of the SALL gene family. SALL4 contains a zinc finger at its amino (N-) terminus and three clusters of zinc fingers, each coordinating zinc with two cysteines and two histidines (Cys2His2 type), which potentially confer nucleic acid binding activity. Rothmund-Thomson syndrome is thought to be due to mutations in RECQL4, with thumb agenesis being one of the numerous phenotypic manifestations [15]. In Balller-Gerold syndrome, where the underlying genetic defect is unknown, thumb hypoplasia or agenesis may be part of the clinical picture [16]. 

The cause of the unique clinical presentation of the index patient remains unclear as the family history for this thumb aplasia syndrome was negative. There is also no congruence with any of the syndromes associated with thumb agenesis previously reported. However, it can be speculated that it is due to spontaneous aneuploidy, chromosomal macro- or microdeletions, or point mutations in genes involved in thumb development. Since none of the family members were affected, it can also be speculated that phenotypic features other than thumb aplasia are due to a second problem. Intrauterine or perinatal trauma as a cause of thumb aplasia is unlikely as the mother did not report any complications during pregnancy or delivery.

The limitation of the study is that chromosomal analysis had not yet been performed, no muscle biopsy was performed, and the patient did not undergo whole-exome sequencing (WES) to rule out a monogenic disorder.

## Conclusions

This case represents a novel syndrome including epilepsy, myopathy, cognitive impairment, mild dysmorphism, and congenital thumb aplasia. Patients with syndromic or non-syndromic thumb aplasia are rare but require regular monitoring, especially in syndromic forms, in order to adjust therapy in the event of progression.
